# Effect of age on fatty infiltration of supraspinatus muscle after experimental tendon release in rats

**DOI:** 10.1186/1756-0500-4-530

**Published:** 2011-12-12

**Authors:** Mazda Farshad, Carola C Würgler-Hauri, Thomas Kohler, Christian Gerber, Dominique A Rothenfluh

**Affiliations:** 1 Balgrist University Hospital, University of Zürich, Forchstrasse 340, 8008 Zürich, Switzerland; 2 Institute for Biomechanics, ETH Zürich, Wolfgang-Pauli-Strasse 10, 8093 Zürich, Switzerland

## Abstract

**Background:**

Rotator cuff tendon tear is a leading cause for atrophy, fibrosis and fatty infiltration of the rotator cuff muscles. The pathophysiology of fatty muscle infiltration is not well understood. An animal model suited to study cellular and molecular mechanisms would therefore be desirable. While a rat model has been established for chronic rotator cuff tendon pathology, sufficient and easily identifiable fatty infiltration of the muscle has not yet been shown in rats. As younger animals regenerate better, we hypothesized that the absence of a sufficient amount of fatty infiltration in previous experiments was due to the selection of young animals and that older animals would exhibit higher amounts of fatty infiltration after tendon tear.

**Findings:**

The supraspinatus tendon was released using tenotomy in 3 young (6 weeks old) and in 3 aged (24 months old) Sprague Dawley rats (group I and II). Another 3 aged (24 months old) rats underwent sham surgery and served as a control group (group III). In group I and II retraction of the musculotendinous unit was allowed for 6 weeks. All animals were sacrificed 6 weeks after surgery and the supraspinatus muscles were harvested. Each sample was examined for fatty infiltration of the muscle by histological methods and micro-CT. In histology, fat cells were counted with a 10-fold magnification in 6 fields of view twice. An adjusted measurement setup was developed for the use of micro-CT to quantify the absorption coefficient of the muscle as a reciprocal indicator for fatty infiltration, based on the established procedure for quantification of fatty infiltration on CT in humans.

Tenotomy resulted in an insignificant increase of fat cells in histological sections in both, aged and young rats. Micro-CT was able to quantify small differences in the absorption coefficients of muscle samples; the absorption coefficient was 8.1% ± 11.3% lower in retracted muscles (group I and II) compared with the control (group III), indicating a tendency towards a higher amount of intra- and/or extracellular fat. Absorption was 4.28% ± 3.2% higher in aged compared with young muscles; however, this difference could not be confirmed by histology.

**Conclusions:**

Substantial fatty muscle infiltration following chronic retraction after supraspinatus tenotomy could be documented histologically neither in young nor aged rats. Although micro-CT was able to reveal minor differences in absorption between the studied groups, the differences were too small to make the rat supraspinatus model interesting to study fatty infiltration of the chronically retracted muscle.

## Background

Rotator cuff tears are a highly prevalent musculoskeletal disorder [1] resulting in deterioration of the musculotendinous unit, characterized by atrophy, fibrosis and fatty infiltration of the muscle [2]. The amount of fatty infiltration of the muscle increases the older the patient and the more extensive the lesion [3] and is associated with suboptimal healing after repair [4,5] and a high rate of rerupture [6]. Continuous traction of the retracted musculotendinous unit to its original length leads to partial restoration of the structural changes but fails to reverse fatty infiltration [7]. The mechanism of fatty infiltration in retracted musculotendinous units after tendon rupture is not well known. It has been postulated that spaces are created between atrophied muscle fibers in which fat cells can infiltrate as a result of retraction and increased pennation angle of the muscle fibers [8]. Accordingly, an increased expression of adipogenic transcription factors was reported in experimentally induced retracted muscles of previously tenotomized muscolotendineous units [9], which could not be identified as the cause of fatty infiltration, however. Currently, there is a significant lack of understanding of the cellular and molecular mechanisms leading to the aforementioned changes in chronically retracted muscles and no molecular or cellular concept for potential reversal has been proposed. A small animal model in which investigations of cellular and molecular mechanism were feasible is therefore desirable. While there have been efforts to establish a rat model for chronic rotator cuff tears [10-13], development of substantial fatty infiltration as seen in humans, could not yet be established by means of tendon release alone. The effect of age of the animals in a rat rotator cuff model has not been investigated so far and remains unknown. Cellular studies on satellite cell function have shown a diminished muscle regeneration potential in the aging microenvironment in rodents [14]. Therefore, we hypothesized that more fatty infiltration may be seen after retraction of the musculotendinous unit in aged rats, whereas it should not or only be seen to a lesser extent in younger animals. A pilot study with 3 young (6 weeks) and 3 aged (24 months) rats were therefore carried out to investigate whether substantial fatty infiltration can be observed in rodents of different ages.

## Methods

This study has been approved by the review board for animal experiments of the Canton of Zurich. The supraspinatus tendon was released bilaterally by tenotomy in 3 aged (24 months old) and 3 young (6 weeks old) Sprague Dawley rats (group I and II) with a lifespan of 2.5-3.5 years. The tendon was wrapped in a silicon tube to prevent spontaneous scarring or healing. Retraction was ensured by placing the wrapped tendon in the supraspinate fossa. Another 3 aged (24 months old) rats underwent sham surgery and served as a control group (group III). The animals ambulated freely for 6 weeks to allow retraction of the musculotendinous unit in group I and II. A 6 week period for retraction was selected to minimize the danger of spontaneous healing by simultaneously allowing enough time for atrophy of the muscle and possibly fatty infiltration to develop. Barton et al. [10] have shown that after tendon release in rats, muscle mass decreases during the first 4 weeks. Furthermore, in a recent rat model of massive rotator cuff tears, significant atrophy and even some minimal amount of fatty infiltration were observed 6 weeks after tendon release [13]. The animals were sacrificed after 6 weeks and the supraspinatus muscle was harvested. Each sample was divided by half and shock-frozen in liquid nitrogen for further evaluation. One half was used for histological evaluation, whereas the other half was used for measurement of absorption with micro-computed tomography (CT).

### Histological evaluation

Each sample was embedded in paraffin for histological evaluation and stained with Hematoxylin-Eosin. Samples were investigated with an Eclipse E600 microscope (Nikon, Egg, Switzerland) using 10-fold magnification. Six representative fields of view (FOV) were photodocumented. In each FOV, fat cells were counted and summed up. This procedure was performed twice and the mean of the counts was used for all comparisons.

### Micro-computed tomography

The other half of each muscle was thawed to room temperature and absorption was measured with micro-CT. The micro-tomographic imaging system (μCT 40, Scanco Medical AG, Brüttisellen, Switzerland) used in this study, is equipped with a 5 μm focal spot x-ray tube as a source. The x-ray tube was operated at 40 kVp and 180 μA with an integration time set to 200 ms and all projection frames were recorded 2 times and then averaged. Scans were performed at an isotropic, nominal resolution of 20 μm (medium resolution mode). A constrained 3D Gaussian filter (σ = 0.8, support of one voxel) was used to partly suppress the noise in the volumes. Then the absorption coefficient as a reciprocal indicator for fatty infiltration, based on the established procedure for quantification of fatty infiltration by CT in humans [15], was averaged within the muscle.

### Statistical analysis

Statistical analysis was performed using the STATA software (Version 11, StataCorp LP, Texas, USA). The Mann-Whitney test was used for intergroup comparison of not normally distributed data. The level of significance was set to *p *< 0.05.

## Results

### Histology

**Figure 1:**
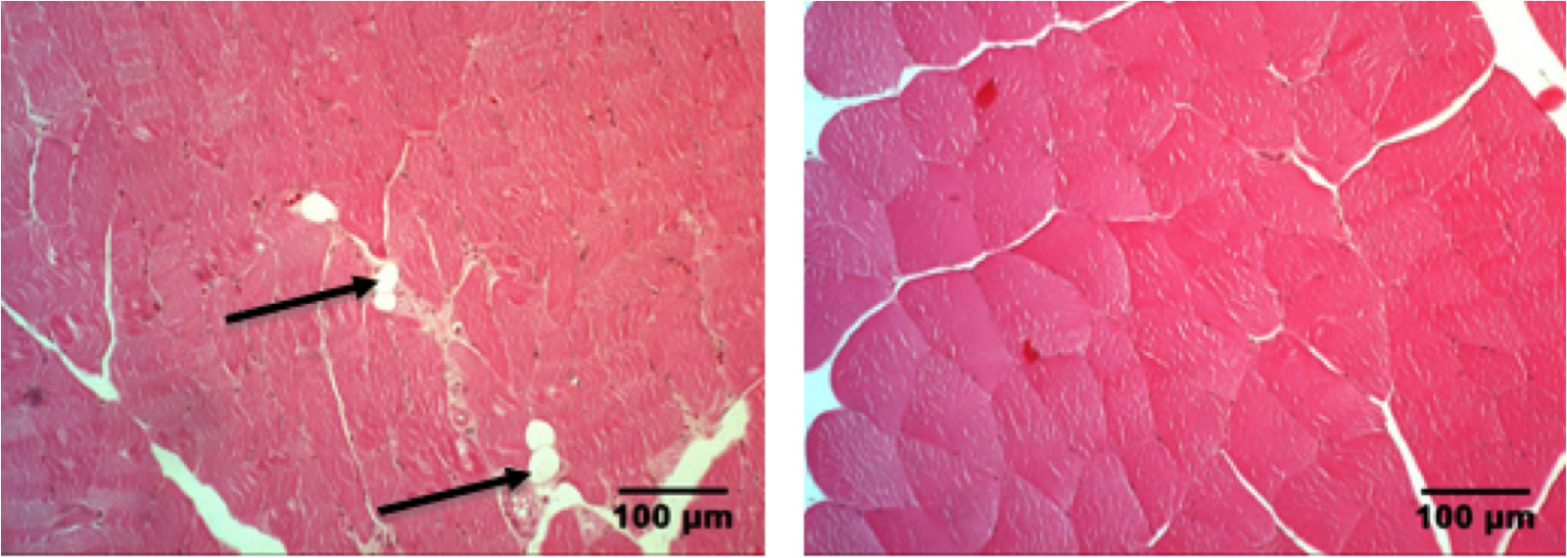
**Fat cells in histological sections**. Fat cells (arrows) were found 6 weeks after tendon release in muscles of aged animals (left) in a small amount and less frequently in the control group that did undergo sham surgery (right).

Tenotomy resulted in a slight increase in fat cell count in aged rats compared to the sham group (Figure 1). The mean count of fat cells in 6 FOV was 4.3 ± 0.8 and 2.2 ± 0.6 in tenotomized muscle of aged rats (group I) and the sham group (III) (*p *= 0.046), respectively. The mean count of fat cells in the muscles of young rats (group II) was 2.5 ± 1. There was no significant difference in the amount of fat cells between tenotomized (group I and II) and sham surgery group (group III) (*p *= 0.145). Furthermore, there was no significant age-dependent difference in fat cell count (*p *= 0.0765). Tenotomy seems to have a slight effect on the amount of fat cells in aged rats but not in younger animals.

### Micro-CT analysis

**Figure 2:**
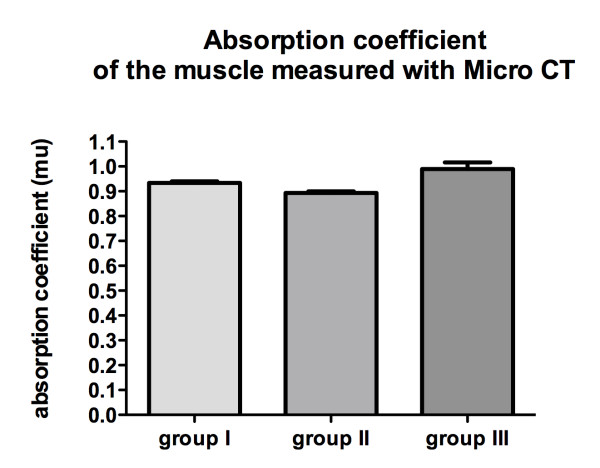
**Micro-CT values**. The absorption coefficient of the muscle measured by micro-CT in mu (1/mm) as a reciprocal indicator for fatty infiltration was higher in group I (0.93 ± 0.01, aged rats, tenotomy) than group group II (0.89 ± 0.01, young rats, tenotomy) but lower than in group III (0.99 ± 0.05, aged rats, sham surgery).

Differences in fat content was further quantified using micro-CT. Lower absorption coefficients are indicative for higher fat content. In aged rats, there was a tendency toward lower absorption in group I (0.93 ± 0.01) compared with group III (0.99 ± 0.05), which was not significant however (*p *= 0.105). Muscle samples of young animals showed an absorption coefficient of 0.89 ± 0.01, which is slightly lower than in the aged group I (*p *= 0.043). The absorption coefficient of muscle samples was 0.96 ± 0.04 in groups I and II combined, which is 8.1% ± 11.3% lower in retracted muscles compared to the sham group (group III) being 0.99 ± 0.05 (*p *= 0.035) (Figure 2).

## Discussion

Substantial fatty infiltration, sufficiently to make the rat rotator cuff model interesting for investigations of fatty infiltration, has not been shown in any rodent model so far. While in humans a degenerated muscle after chronic rotator cuff tear can show fatty infiltration up to more than 50% of the muscle volume (Goutallier Grade IV [15]), the percentage of fat in degenerated muscles in rats seems to be at best around 1% [13,16].

Up to date, the effect of age of the animal in the rat rotator cuff model remained unknown; Based on observations of cellular studies focusing on satellite cell function, which reported a diminished regeneration potential of muscles in the aging microenvironment in rodents [14], we hypothesized that fatty infiltration may be seen after retraction of the musculotendinous unit of the supraspinatus in aged rats. With the expectation to only find small differences in histological sections, we employed micro-CT to assess small differences in absorption as a reciprocal indicator for fatty infiltration in analogy to the well established method used for quantification of fatty infiltration in humans using CT [15].

We found very small differences in the amount of fat between the investigated groups both in histological sections as well as micro-CT. However, the findings were not concordant. The tenotomy groups combined (groups I and II, aged and young) did not demonstrate significant differences in fat cell count compared to the sham group, but micro-CT revealed a higher fat content. In aged rats, tenotomy resulted in a slight increase in fat cell count, which is contradicting the higher absorption measured by micro-CT in aged muscles compared to the group of young animals (group II). It would be expected, however, that both methods yield concordant findings pointing toward relevant fatty infiltration in a valid model. Thus, the aging microenvironment is unlikely to contribute to fatty infiltration in rats as evidenced by the unexpected higher absorption, i.e. less fat, of aged muscles.

As this investigation was planned as a pilot study, the sample-size per group is small. While statistical significance could be reached at least in part, the differences of the results were smaller than expected and the model is therefore not considered to be relevant for fatty infiltration. Considering the small size of the groups and small intergroup-differences in fatty infiltration, animal-to-animal variability may have contributed to the discordant findings as well as methodological differences as micro-CT detects intracellular and extracellular fat, whereas in histology only extracellular fat cells were counted. Our findings indicate that a rat model of fatty infiltration upon tendon retraction of the rotator cuff may not provide sufficient information on the extent of fatty infiltration that could be expected between groups using micro-CT. While differences in the absorption coefficients were detected, tenotomy resulted only in 8.1% lower absorption, i.e. more fat content, in aged and young rats combined compared to the sham group. In order to carry out studies on the pathomechanism of fatty infiltration, an animal model with established relevant differences in fat amount in retracted compared to non-retracted muscles, such as the sheep shoulder model [17], seems required. From a methodological point of view, micro-CT with the absorption coefficient as a reciprocal indicator for fatty infiltration, based on quantification of fatty infiltration in the human rotator cuff [15], yielded values with a narrow range of intra-group variances, indicating the accuracy of the method.

Due to the very small amounts of fatty infiltration, an enormous number of animals would be needed in order to reach statistical significance for the use of the rat supraspinatus tendon release model for investigations of fatty infiltration. According to the results of this pilot study, a post-hoc sample size calculation shows that 1301 animals would be needed per group in order to yield a significant difference of the absorption coefficient of the muscle of aged rats undergoing tenotomy versus those undergoing sham surgery. Furthermore, we needed 6 FOV in order to find fat cells in this study and found the most amount, namely a mean of 4.3 ± 0.8 cells, in the aged rats muscle versus 2.2 ± 0.6 cells in the sham surgery control group. This seems to be a very small difference and as such, it might have arisen from measurement errors or by chance.

In conclusion, a sufficient amount of fatty infiltration following chronic retraction after tenotomy of the supraspinatus muscle of aged rats could not be conclusively shown in the present model by histology. Although micro-CT was able to reveal differences in absorption after tenotomy, the differences seem to be too small to make the rat supraspinatus tenotomy model relevant for investigations of fatty infiltration of the chronically retracted muscle even with the use of aged animals.

## Competing interests

The authors declare that they have no competing interests.

## Authors' contributions

MF was involved in conception and design and acquisition of data and analysis and interpretation of data, as well as drafting the manuscript. CCWH and TK were involved in conception and design and acquisition of data and analysis and interpretation of data and drafting part of the manuscript. CG was involved in conception and design of the study, interpretation of data and revising the manuscript critically for important intellectual content. DAR was involved in conception and design and acquisition of data and analysis and interpretation of data, as well as revising the manuscript critically for important intellectual content. All authors read and approved the final manuscript.

## References

[B1] Roquelaure Y Ha C Leclerc A Touranchet A Sauteron M Melchior M Imbernon E Goldberg M Epidemiologic surveillance of upper-extremity musculoskeletal disorders in the working population Arthritis Rheum 2006 55 5 765 778 10.1002/art.22222 17013824

[B2] Gerber C Meyer DC Schneeberger AG Hoppeler H von Rechenberg B Effect of tendon release and delayed repair on the structure of the muscles of the rotator cuff: an experimental study in sheep J Bone Joint Surg Am 2004 86-A 9 1973 1982 15342760 10.2106/00004623-200409000-00016

[B3] Melis B Nemoz C Walch G Muscle fatty infiltration in rotator cuff tears: descriptive analysis of 1688 cases Orthop Traumatol Surg Res 2009 95 5 319 324 10.1016/j.otsr.2009.05.001 19586809

[B4] Goutallier D Postel J Lavau L Bernageau J Influence de la dégénérescence musculaire du supraspinatus et de l'infraspinatus sur le prognostic des réparations chirurgicalesde la couffe des rotateurs Acta Orthop Belg 1998 64 Suppl II 42 45 9922528

[B5] Goutallier D Postel JM Gleyze P Leguilloux P Van Driessche S Influence of cuff muscle fatty degeneration on anatomic and functional outcomes after simple suture of full-thickness tears J Shoulder Elbow Surg 2003 12 6 550 554 10.1016/S1058-2746(03)00211-8 14671517

[B6] Goutallier D Postel JM Bernageau J Lavau L Voisin MC Fatty infiltration of disrupted rotator cuff muscles Rev Rhum Engl Ed 1995 62 6 415 422 7552205

[B7] Gerber C Meyer DC Frey E von Rechenberg B Hoppeler H Frigg R Jost B Zumstein MA Neer Award 2007: Reversion of structural muscle changes caused by chronic rotator cuff tears using continuous musculotendinous traction. An experimental study in sheep J Shoulder Elbow Surg 2009 18 2 163 171 10.1016/j.jse.2008.09.003 19095462

[B8] Meyer DC Hoppeler H von Rechenberg B Gerber C A pathomechanical concept explains muscle loss and fatty muscular changes following surgical tendon release J Orthop Res 2004 22 5 1004 1007 10.1016/j.orthres.2004.02.009 15304272

[B9] Frey E Regenfelder F Sussmann P Zumstein M Gerber C Born W Fuchs B Adipogenic and myogenic gene expression in rotator cuff muscle of the sheep after tendon tear J Orthop Res 2009 27 4 504 509 10.1002/jor.20695 18932240

[B10] Barton ER Gimbel JA Williams GR Soslowsky LJ Rat supraspinatus muscle atrophy after tendon detachment J Orthop Res 2005 23 2 259 265 10.1016/j.orthres.2004.08.018 15734235

[B11] Schneeberger AG Nyffeler RW Gerber C Structural changes of the rotator cuff caused by experimental subacromial impingement in the rat J Shoulder Elbow Surg 1998 7 4 375 380 10.1016/S1058-2746(98)90026-X 9752647

[B12] Soslowsky LJ Carpenter JE DeBano CM Banerji I Moalli MR Development and use of an animal model for investigations on rotator cuff disease J Shoulder Elbow Surg 1996 5 5 383 392 10.1016/S1058-2746(96)80070-X 8933461

[B13] Liu X Manzano G Kim HT Feeley BT A rat model of massive rotator cuff tears J Orthop Res 2011 29 4 588 595 10.1002/jor.21266 20949443

[B14] Conboy IM Conboy MJ Wagers AJ Girma ER Weissman IL Rando TA Rejuvenation of aged progenitor cells by exposure to a young systemic environment Nature 2005 433 7027 760 764 10.1038/nature03260 15716955

[B15] Goutallier D Postel JM Bernageau J Lavau L Voisin MC Fatty muscle degeneration in cuff ruptures. Pre- and postoperative evaluation by CT scan Clin Orthop Relat Res 1994 304 78 83 8020238

[B16] Buchmann S Walz L Sandmann GH Hoppe H Beitzel K Wexel G Battmann A Vogt S Hinterwimmer S Imhoff AB Rotator cuff changes in a full thickness tear rat model: verification of the optimal time interval until reconstruction for comparison to the healing process of chronic lesions in humans Arch Orthop Trauma Surg 2011 131 3 429 435 10.1007/s00402-010-1246-5 21190029

[B17] Gerber C Schneeberger AG Perren SM Nyffeler RW Experimental rotator cuff repair. A preliminary study J Bone Joint Surg Am 1999 81 9 1281 1290 10505524 10.2106/00004623-199909000-00009

